# A fluorescence-based assay for *Trichomonas vaginalis* drug screening

**DOI:** 10.1186/s13071-023-05919-6

**Published:** 2023-09-18

**Authors:** Qianqian Chen, Jingzhong Li, Zhensheng Wang, Wei Meng, Heng Wang, Zenglei Wang

**Affiliations:** 1 NHC Key Laboratory of Systems Biology of Pathogens, Institute of Pathogen Biology, Chinese Academy of Medical Sciences and Peking Union Medical College, Beijing, 100005 People’s Republic of China; 2 NHC Key Laboratory of Echinococcosis, Prevention and Control, Tibet Autonomous Region Center for Disease Control and Prevention, 21# Linkuo North Road, Chengguan District, Lhasa, 850000 People’s Republic of China; 3 Department of Microbiology and Parasitology, Institute of Basic Medical Sciences, Chinese Academy of Medical Sciences and School of Basic Medicine, Peking Union Medical College, Beijing, 100005 People’s Republic of China

**Keywords:** *Trichomonas vaginalis*, Drug resistance, High-throughput, Assay

## Abstract

**Background:**

The emergence and spread of drug resistance in *Trichomonas vaginalis* parasites has become an important concern in trichomoniasis treatment. Fast and reliable growth assessment is critical for validating in vitro drug susceptibility and high-throughput screening of newly developed drugs.

**Methods:**

Modified media without yeast extract were evaluated for their ability to support the growth of *T. vaginalis* parasites. The potential of the nucleic acid-binding dye SYBR Green I for detecting *T. vaginalis* drug resistance was characterized, and seeding parasite concentration and incubation time were optimized. The fluorescence assay based on SYBR Green I was further validated in four *T. vaginalis* isolates with different susceptibilities to the antibiotics metronidazole, tinidazole, ornidazole and secnidazole, and compared with the traditional method that detects minimum lethal concentrations (MLCs).

**Results:**

A modified medium consisting of RPMI 1640 and Tryptone Plus as replacements for yeast extract and tryptone, respectively, in traditional trypticase-yeast extract-maltose (TYM) medium exhibited similar performance as TYM medium in maintaining *T. vaginalis* growth, while it showed much lower background fluorescent signals. The *T. vaginalis* SYBR Green I-based fluorescence (TSF) drug assay was found to have to satisfy one of two conditions to demonstrate the 50% inhibitory concentration of metronidazole for the sensitive isolate TV-334: (i) a seeding density of 3 × 10^4^ parasites/ml and an incubation time of 48 h; or (ii) a seeding density of 1 × 10^4^ parasites/ml and an incubation time of 72 h. Subsequent validation experiments revealed that the 48-h incubation/3 × 10^4^ parasites/ml seeding density condition had a greater sensitivity to detect drug resistance than the 72-h condition. The TSF assay also exhibited high efficiency in identifying parasite drug resistance, as evidenced by its strong correlation with the standard MLC assay results (*P* = 0.003).

**Conclusions:**

This study presents a robust TSF assay that has the potential to facilitate high-throughput, automated in vitro anti-trichomoniasis susceptibility testing for drug resistance monitoring and drug development. In comparison to the standard MLC method, this assay offers the advantages of reduced labor and elimination of subjective examination.

**Graphical Abstract:**

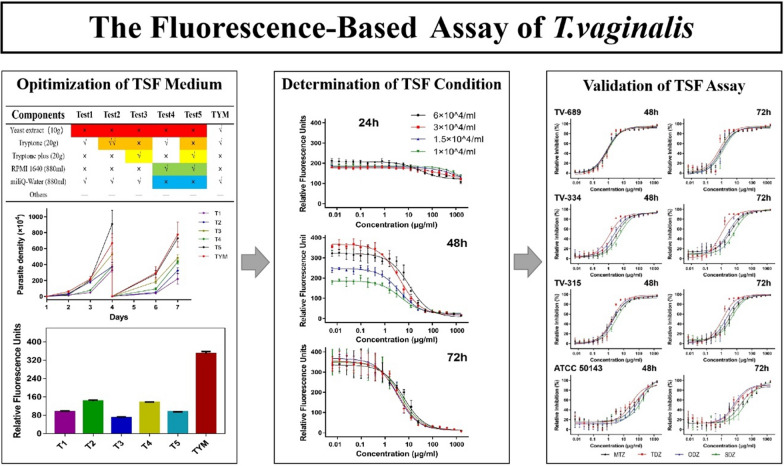

**Supplementary Information:**

The online version contains supplementary material available at 10.1186/s13071-023-05919-6.

## Background

Trichomoniasis, which is the most prevalent non-viral sexually transmitted infection (STI), is caused by *Trichomonas vaginalis*, a flagellated extracellular microbial eukaryote with an anaerobic lifestyle [1]. The WHO reports an annual incidence of 156 million cases [1, 2], but trichomoniasis is not a reportable disease and, therefore, the actual number might be underestimated since wet preparation microscopy is the standard diagnostic test and no formal surveillance system is in place [3]. *Trichomonas vaginalis* infection induces significant health sequelae in both women and men. Recent studies have highlighted its epidemiological association with a variety of health conditions, including infertility, adverse birth outcomes, cervical neoplasia, pelvic inflammatory disease, urethritis and prostate cancer. In particular, *T. vaginalis* infections amplifies the acquisition and transmission of human immunodeficiency virus (HIV) [4–7]. Since the 1950s, 5-nitroimidazole-based drugs have been the first-line therapeutic approach for trichomoniasis; of these, metronidazole (MTZ) and tinidazole (TDZ) are the two most frequently prescribed therapeutic regimens. However, persistent and recurrent infections have become a great concern due to increasing drug resistance [8]. MTZ-resistant isolates have been reported worldwide, with the prevalence ranging from 2.2% to 9.6% across different regions [9–15]. The emergence of TDZ resistance has also been observed, with a reported frequency of 2.0% among the general population of South Africa [16]. Given the lack of clarity regarding the resistance mechanism and the absence of improved therapeutic options, microbiological surveillance through antimicrobial susceptibility testing is crucial for monitoring the spread of drug resistance and assessing the risk of treatment failure. Furthermore, dependence on a single class of antimicrobial drugs underscores the urgent need for screening and developing alternative agents beyond of the 5-nitroimidazoles.

For several decades, the assessment of anti-trichomoniasis drug sensitivity has incorporated the in vitro measurement of the minimum lethal concentration (MLC). MLC is defined as the lowest concentration of a drug at which no motile or viable trichomonads can be detected through culture under either aerobic or anaerobic conditions [17, 18]. However, this method has become increasingly impractical due to increasing testing volume, primarily because it is labor-intensive and strongly subjective: it requires manual observation of live parasites in each well of a microtiter plate under the microscope after a 48-h incubation with drugs, resulting in significant inputs of time and effort. Moreover, the exposure of the trophozoite stage of *T. vaginalis* to stress conditions, such as drug treatment, might induce the formation of pseudocysts in vitro [19], making it challenging to differentiate live and dead parasites. Therefore, there is a pressing need to develop a new approach that is both reliable and feasible for a high-throughput assay.

SYBR Green I is an asymmetrical cyanine dye that has been used as a substitute for ethidium bromide due to its ability to interact with nucleic acids [20, 21]. A significant fluorescence enhancement can be detected when it binds directly to DNA or RNA, and the fluorescence intensity can thus reflect the amount of nucleic acids in the sample. This property makes SYBR Green I an ideal tool to quantify the growth of organisms under specific conditions, such as drug pressure, an approach which has been successfully employed in malaria high-throughput drug assays [22–24]. Consequently, SYBR Green I has the potential for adoption in the protistic *T. vaginalis* parasite sensitivity assay.

In this study, we report a cost-effective and time-saving in vitro* T. vaginalis* SYBR Green I-based fluorescence (TSF) assay that is suitable for high-throughput testing of *T. vaginalis* drug susceptibility as well as screening for new anti-trichomoniasis drugs.

## Methods

### *Trichomonas vaginalis* cultivation and reagents

The MTZ-resistant *T. vaginalis* isolate ATCC 50143 was obtained from the American Type Culture Collection (ATCC, Manassas, VA, USA). Three antimicrobial drug-sensitive *T. vaginalis* isolates, TV-689, TV-315 and TV-334, were isolated in 2012 and stored in liquid nitrogen until use. The antibiotics MTZ and ornidazole (ODZ) were purchased from Sigma-Aldrich (St. Louis, MO, USA, and the antibiotics TDZ and secnidazole (SDZ) were purchased from J&K Scientific Company (Shanghai, China). Tryptone and Tryptone Plus were obtained from Merck LLC (Shanghai, China), and yeast extract and SYBR Green I nucleic acid staining dye (10,000× stock concentration) were obtained from Thermo Fisher Scientific (Waltham, MA, USA). Roswell Park Memorial Institute (RPMI) 1640 medium (without phenol red) was purchased from Procell Life Science & Company (Wuhan, China). The lysis buffer was prepared using Tris–HCl (20 mM, PH 7.5), ethylene diamine tetraacetic acid (EDTA; 5 mM), Triton X-100 (0.08%) and saponin (0.008%). Diamond’s trypticase, yeast extract and maltose (TYM) medium was prepared as previously reported [23, 24].

The parasites were thawed and maintained in TYM medium supplemented with 10% heat-inactivated fetal bovine serum, penicillin (100 U/ml) and streptomycin (0.1 mg/ml) at 37 °C in either a regular incubator or a CO_2_ incubator according to requirements. Five passages through complete medium containing an additional 20 μg/ml of tetracycline were carried out to inhibit the growth of *Mycoplasma* spp. that might be associated with *T. vaginalis* before optimization of the medium for the drug assays. Daily counting and morphological examination of the parasites were performed to ensure proper culture maintenance.

### Determination of *T. vaginalis* SYBR Green I-based fluorescence culture medium

Prior to the test, parasites of strain TV-334 were conditioned to their respective test medium by three to four passages. The test media were based on TYM medium and modified with different components, including yeast extract, tryptone, Tryptone Plus and RPMI 1640 medium as variables (Table 1).

**Table 1:** Test media with different components

Components (per liter)	Test media					
	T1	T2	T3	T4	T5	TYM
Yeast extract (10 g)	×	×	×	×	×	√
Tryptone (20 g)	√	√√	×	√	×	√
Tryptone Plus (20 g)	×	×	√	×	√	×
RPMI 1640 (880 ml)	×	×	×	√	√	×
MilliQ-water (880 ml)	√	√	√	×	×	√
Common components^a^	√	√	√	√	√	√

The symbol “√” indicates the inclusion of a component in the medium; the symbol “×” implies the exclusion of a component; the symbol “√√” specifies the inclusion of double the volume of a component

^a^Common components of the medium included 5 g of maltose, 1 g of l-cysteine hydrochloride, 0.2 g of ascorbic acid, 0.8 g of KH_2_PO_4_, 0.8 g of K_2_HPO_4_, penicillin sodium at 100 U/ml, streptomycin sulfate at 100 μg/ml and 100 ml of fetal bovine serum

For the test assays, parasites at a density of 1 × 10^4^ parasites/ml were cultured in 24-well plates, with each well containing 2 ml of medium; each medium was tested in three replicate wells. Every 24 h, the concentration of *T. vaginalis* was determined by hemocytometer counts, and 200 μl of completely mixed culture from each well was then transferred to a new well with 1.8 ml fresh medium for continuous culture. At day 4, the densities of parasites in each culture were reduced to 1 × 10^4^ parasites/ml again to avoid overgrowth.

### Assessment of fluorescence linearity

The SYBR Green I fluorescence linearity of parasite density from 0 to 1 × 10^7^ cells/ml was assessed as previously described, with modification [24]. Briefly, triplicate wells of TV-334 were serially diluted with fresh medium to a final volume of 100 μl with parasite densities ranging from 0 to 1 × 10^7^ cells/ml. After a freeze–thaw cycle, 100 μl of lysis buffer containing SYBR Green I (1× final concentration) was added directly to the plates and gently mixed using a multichannel pipette. The plates were then incubated in the dark at 37 °C for 1 h, and the fluorescence was determined. The background fluorescence values for the medium were subtracted , and the counts were plotted and analyzed by linear regression to determine the goodness of fit (*r*^2^ value) using GraphPad Prism 7 (GraphPad Software, San Diego, CA, USA).

### Investigation of conditions for the TSF assay

The TV-334 cultures were prepared with conditioned medium and diluted to concentrations of 6 × 10^4^, 3 × 10^4^, 1.5 × 10^4^ and 1 × 10^4^ parasites/ml. MTZ was dissolved in the culture medium containing 0.3% DMSO to make a MTZ stock solution of 32 mg/ml. This stock solution was subsequently further diluted to reach a concentration of 3200 μg/ml for the assay. The assay was performed using round-bottom 96-well plates. The outer round wells (rows 1 and 8, columns 1 and 12) were filled with phosphate-buffered saline to prevent the edge effect. For each of the tested cultures, 100 μl of culture was dispensed into every inner well, except for those in column 2, which were filled with 100 μl of culture at 2× the parasite density. The MTZ solution was dispensed into wells of column 2 and serially diluted to yield concentrations ranging from 1600 to 0.0061 μg/ml. The plates were incubated at 37 °C for 24, 48 and 72 h. After incubation, 100 μl of lysis buffer with SYBR Green I was added to each well, mixed and incubated in the dark at 37 °C for 3 h. The fluorescence was measured with a Fluostar Optima microplate fluorometer (BMG Labtech, Ortenberg, Germany) at 485 nm excitation and 528 nm emission. The 50% inhibition concentration (IC_50_) values were analyzed with GraphPad Prism 7 software (GraphPad Software). Two technical replicates and three biological replicates were performed.

### Validation of capacity of TSF assay

Four *T. vaginalis* isolates (TV-689, TV-334, TV-315, ATCC 50143) were diluted to seeding concentrations of 1 × 10^4^ and 3 × 10^4^ parasites/ml, respectively, and incubated for 48 or 72 h to assess their susceptibility against MTZ, TDZ, ODZ and SDZ by the same procedure described above. For comparison purposes, the in vitro susceptibilities of these isolates against those four drugs was determined using the standard MLC method as previously reported [10]. Each plate was independently inspected by two individuals; if the results were inconsistent, microscopic examination was conducted by a third individual.

### Statistical analysis

The consistency between the drug sensitivity data obtained from the TSF assay and the MLC method was analyzed by Kendall rank correlation through the SPSSPRO platform [25].

## Results

### Optimization of culture medium

**Fig. 1:**
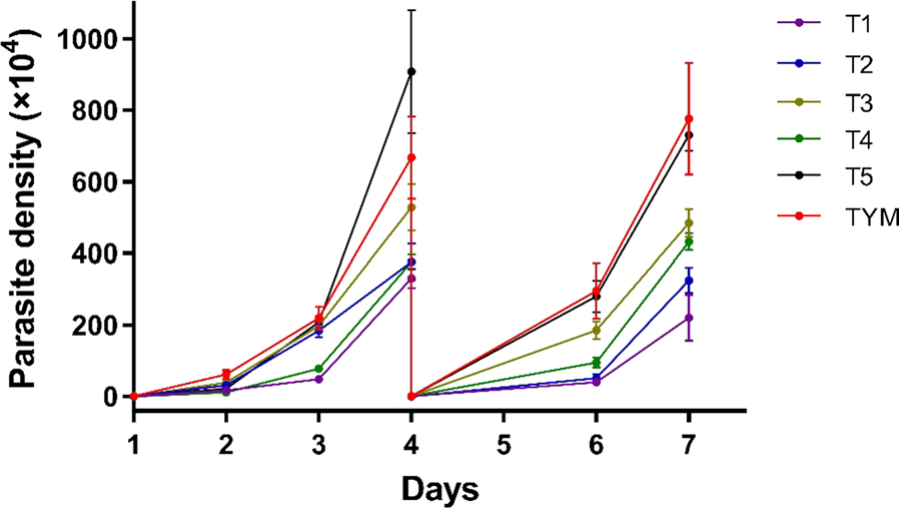
Evaluation of the effectiveness of media with different components in supporting of *Trichomonas vaginalis* growth. Growth curves were generated by daily counting of parasite numbers for 7 consecutive days. The density was reduced to 1 × 10^4^ parasites/ml on day 4 to avoid overgrowth. T1–T5, Test media T1–T5 (see section Optimization of culture medium for details); TYM, Trypticase-yeast extract-maltose

**Fig. 2:**
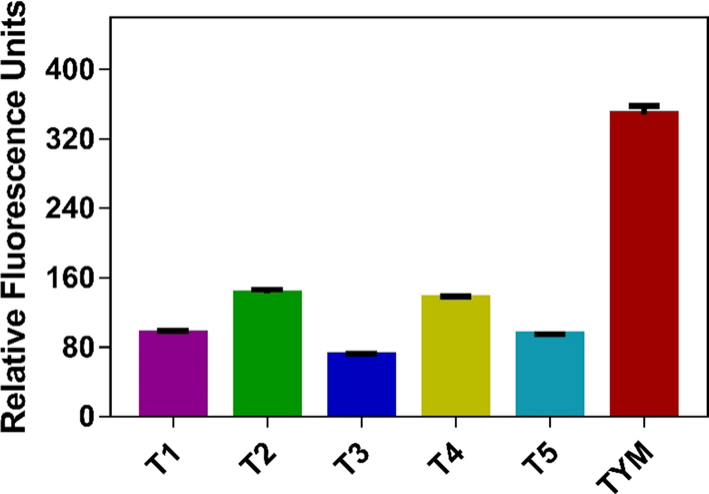
Background fluorescence signals of the tested media. The histogram was generated based on three biological replicates. T1–T5, Test media T1–T5 (see section Optimization of culture medium for details); TYM, trypticase-yeast extract-maltose (medium)

Given that the yeast extract in traditional Diamond’s TYM medium contains > 10% nucleotide content [26, 27], which would interact with SYBR Green I and excite fluorescence, we evaluated the efficacy of five modified media without yeast extract in maintaining *T. vaginalis* growth, using TYM as the reference medium. Test medium 1 (T1) excluded yeast extract based on TYM, while test medium 2 (T2) comprised double amounts of tryptone compared to T1. Tryptone Plus was utilized to replace tryptone in test medium 3 (T3). Test medium 4 (T4) substituted yeast extract with RPMI 1640, and test medium 5 (T5) incorporated both RPMI 1640 and Tryptone Plus in the system (Table 1, Table S1). As illustrated in Fig. 1, T5 showed a similar capability to the traditional TYM medium for continuous support of parasite growth while exhibiting a significantly lower fluorescence background (Fig. 2, Table S2). These characteristics bestowed T5 with properties suitable for utilization in the SYBR Green I-based assay. However, due to the slow growth of parasites under the aerobic condition in both TYM and the five test media (Additional file 1: Figure S1, Table S3), the assay could only be performed under the anaerobic condition.

### Linearity of SYBR Green I fluorescence over *T. vaginalis* density

**Fig. 3:**
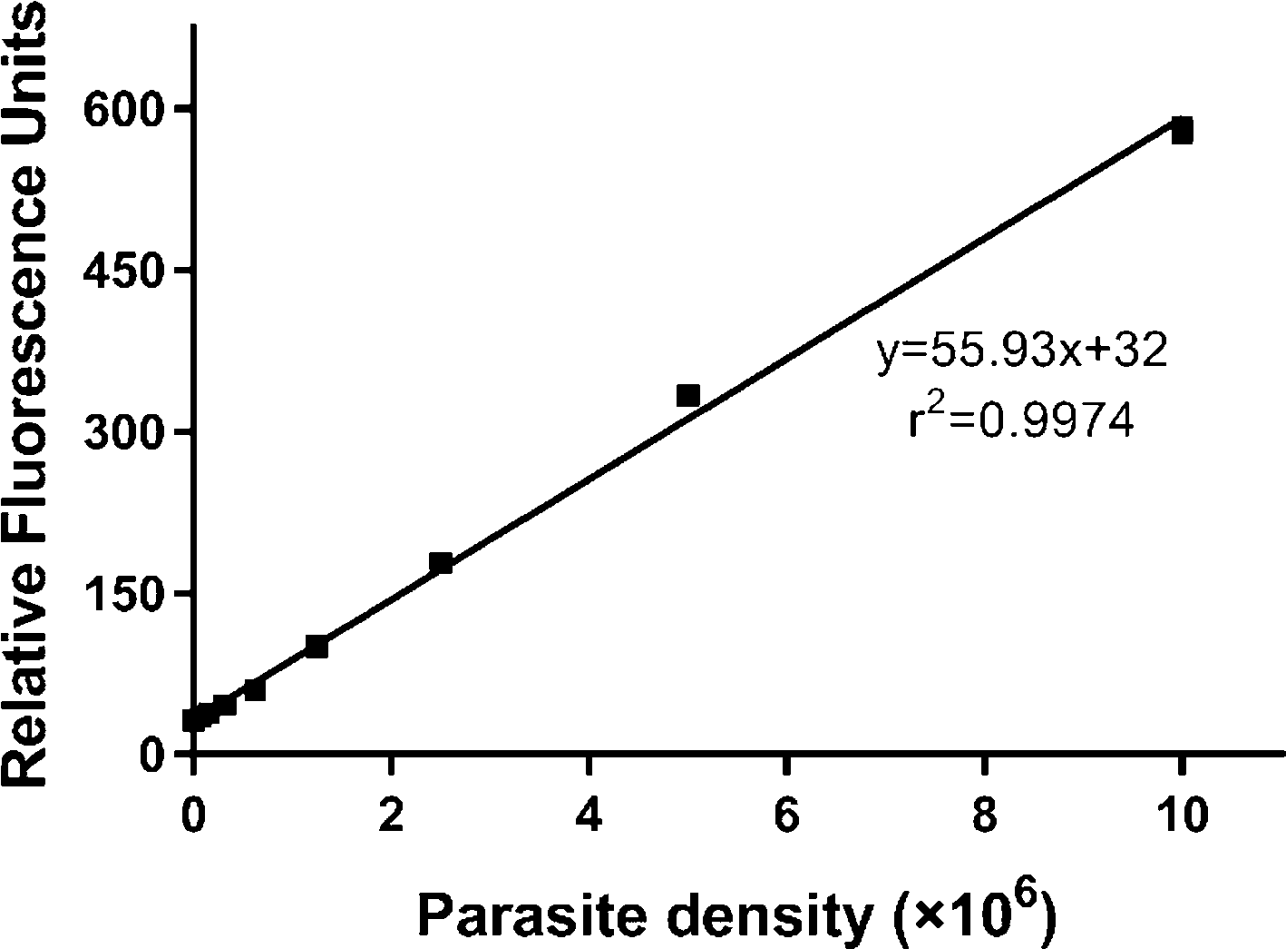
Assessment of fluorescence linearity. The relative fluorescence units were plotted against the density of *T. vaginalis*, and the data were presented as the mean of triplicate samples. TSF, *T. vaginalis* SYBR Green I-based fluorescence; TYM, trypticase-yeast extract-maltose (medium)

The fluorescence linearity of SYBR Green I over a range of known *T. vaginalis* parasite densities from 0 to 1 × 10^7^ cells/ml was determined by microscopic examination of the TV-334 isolate and measurement of fluorescence. As expected, a linear relationship between *T. vaginalis* parasite densities and SYBR Green I fluorescence was verified (*r*^2^ = 0.9974) (Fig. 3, Table S4), indicating that fluorescence intensity was a good reflection of the density of *T. vaginalis* parasites.

### Evaluation of TSF assay conditions

**Fig. 4:**
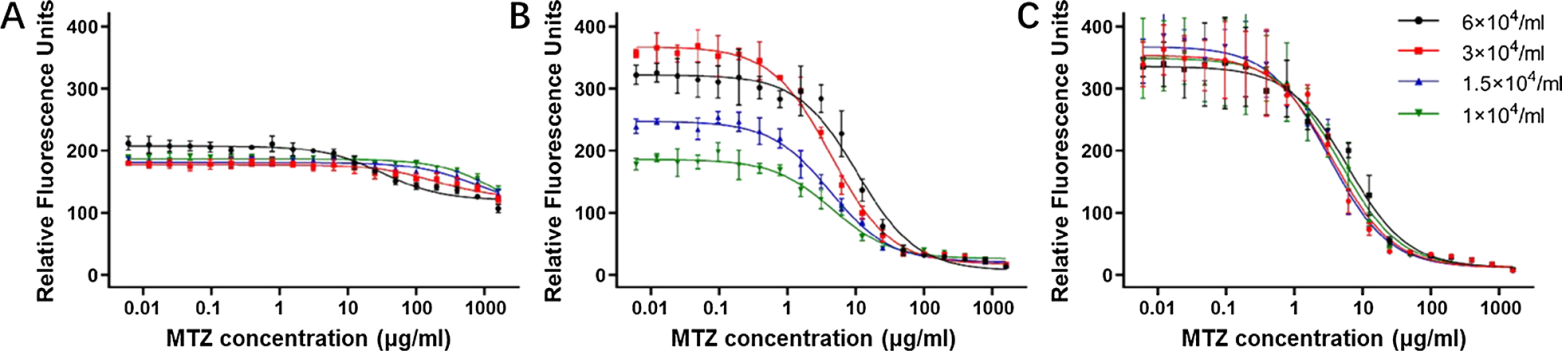
Comparison of drug dose–response curves under different seeding concentrations and incubation time. a, b, c Test performance under incubation times of 24, 48 and 72 h, respectively. Error bars indicate the standard deviation. MTZ, Metronidazole

To determine the optimal initial parasite concentrations and incubation time for the TSF assay, cultures of TV-334 containing 6 × 10^4^, 3 × 10^4^, 1.5 × 10^4^ and 1 × 10^4^ parasites/ml were treated with MTZ for 24, 48 and 72 h in parallel. Each experimental group was subjected to a serial dilution of MTZ from 1600 to 0.0061 μg/ml (Table S5). As shown in Fig. 4a, the optical density values after 24 h of incubation were consistently low across all culture conditions, implying incomplete growth of *T. vaginalis.* Sigmoidal curves appeared for all groups after 48 or 72 h of incubation (Fig. 4b, c, respectively). However, notable variations were observed in the curves of different cultures with varying concentrations of parasites after 48 h of incubation, while relatively consistent patterns were observed 72 h after MTZ treatment. Microscopic inspection revealed dead cells in the control wells of all parasite concentrations in the 72-h group due to excessive proliferation of *T. vaginalis*, with the exception of the culture well with the lowest concentration of parasites (1 × 10^4^ parasites/ml). A similar phenomenon was identified in the wells with the highest concentration in the 48-h group, while cultures with low concentrations of 1 × 10^4^ and 1.5 × 10^4^ parasites/ml in this group did not exhibit sufficient replication. Therefore, we considered the appropriate TSF assay conditions to be a seeding concentration of 3 × 10^4^ parasites/ml with a 48-h incubation period, and a seeding concentration of 1 × 10^4^ parasites/ml with a 72-h incubation period, and that these assays needed to be further validated.

### Validation of TSF assay using isolates with different drug susceptibilities

**Fig. 5:**
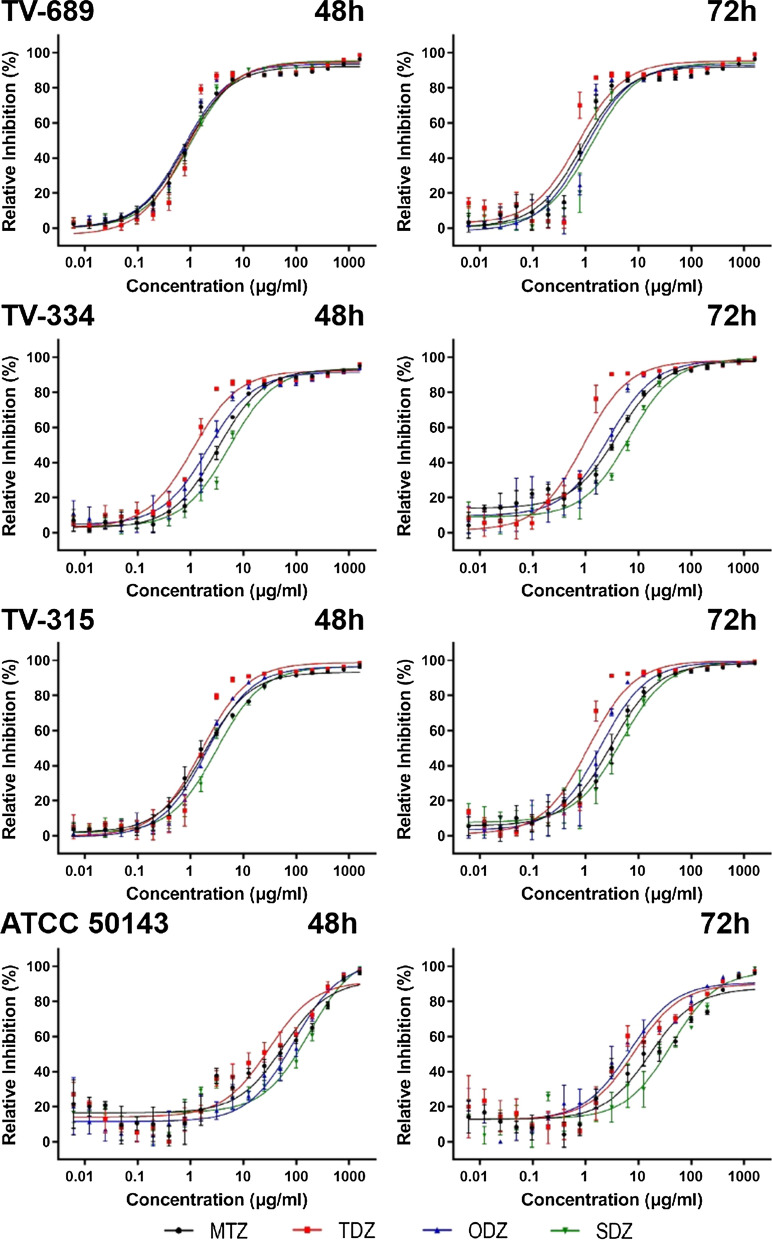
Validation of assay conditions utilizing isolates with varying responses against four antimicrobial drugs. The left panel shows the results of the TSF assay performed with an initial parasite seeding concentration of 3 × 10^4^ parasites/ml and an incubation time of 48 h. The right panel shows the results of the TSF assay with an initial seeding concentration of 1 × 10^4^ parasites/ml and an incubation time of 72 h. Error bars indicate the standard deviation. ACC 50143, MTZ-resistant *T. vaginalis* strain; MTZ, metronidazole; ODZ, ornidazole; SDZ, secnidazole; TDZ, tinidazole; TV-689, TV-315, TV-334, *T. vaginalis* strains

To validate these two assay conditions, we evaluated four isolates with varying drug susceptibilities against four anti-trichomoniasis drugs: MTZ, TDZ, ODZ and SDZ (Table S6). Results from the standard MLC method were employed as reference. To our surprise, the IC_50_ values of sensitive isolates remained relatively consistent under both conditions; in contrast, significant differences were detected in the results for the resistant ATCC 50143 isolate (Fig. 5; Table 2). The TSF assay carried out under the 48-h incubation condition exhibited a greater sensitivity to the determination of drug resistance compared to the 72-h condition, suggesting that the seeding concentration of 3 × 10^4^ parasites/ml and an incubation time of 48 h are suitable conditions for the TSF assay. Under these latter conditions, the assay results demonstrated a significant consistency with the data from the MLC method, as evidenced by the Kendall’s *W* coefficient of 0.562 and a *P* value of 0.003 (Table 3).

**Table 2:** The 50% inhibitory concentration values of the different isolates under the two assay conditions, and minimum lethal concentration results

*Trichomonas vaginalis* isolate	Test drugs	IC_50_ (μg/ml) (Mean ± SD)		MLC (μg/ml)
		48-h incubation period	72-h incubation period	
TV-689	MTZ	0.79 ± 0.07	0.86 ± 0.07	1.56
	TDZ	0.83 ± 0.07	0.73 ± 0.08	0.78
	ODZ	0.76 ± 0.01	0.98 ± 0.10	1.56
	SDZ	0.98 ± 0.06	1.28 ± 0.25	3.13
TV-334	MTZ	3.56 ± 0.39	4.66 ± 0.47	12.5
	TDZ	1.11 ± 0.09	0.90 ± 0.07	1.56
	ODZ	2.16 ± 0.34	2.77 ± 0.33	3.13
	SDZ	5.39 ± 0.50	6.73 ± 0.92	12.5
TV-315	MTZ	2.30 ± 0.32	2.92 ± 0.34	6.25
	TDZ	1.67 ± 0.12	1.14 ± 0.02	1.56
	ODZ	2.01 ± 0.10	1.95 ± 0.21	3.13
	SDZ	3.13 ± 0.22	4.70 ± 0.64	6.25
ATCC50143	MTZ	67.1 ± 16.1	20.3 ± 6.26	200
	TDZ	30.5 ± 14.7	8.44 ± 0.19	100
	ODZ	90.4 ± 22.0	6.84 ± 0.44	200
	SDZ	176 ± 14.4	42.3 ± 6.89	400

*IC*_*50*_ 50% Inhibitory concentration,* MLC* minimum lethal concentration,* MTZ* metronidazole,* ODZ* ornidazole,* SDZ* secnidazole,* TDZ* tinidazole

**Table 3:** Comparison of consistency of results from *T. vaginalis* SYBR Green I-based fluorescence and minimum lethal concentration assays

Data	Kendall's test			
	Mean rank	Median	Kendall’s *W* coefficient	*P*
TSF	1.125	2.228	0.562	0.003***
MLC	1.875	4.69		

^***^Significance at* P* < 0.01

*MLC* Minimum lethal concentration,* TSF* *T. vaginalis* SYBR Green I-based fluorescence

## Discussion

The need to monitor drug resistance in *T. vaginalis* and to screen new antimicrobial drugs is increasing and, therefore, the development of an accurate high-throughput approach is important. In this study, we validated the suitability of a SYBR green I-based fluorescence assay for assessing drug susceptibility in *T. vaginalis* and developed a time- and labor-saving TSF assay method that is feasible for high-throughput testing.

The SYBR Green I assay was initially developed as a replacement for the [3H]hypoxanthine incorporation assay, which was the previous standard for in vitro antimalarial drug efficacy testing [24]. Over time, this method came to be considered as the “gold standard” for assessing drug resistance in malaria parasites. The advantages of this method, including time- and labor-saving features, have led to its widespread adoption in other protistic parasites. The application of the SYBR green I assay in drug screening against *Babesia *spp., a family of blood parasites similar to *Plasmodium *spp., has been evaluated [28] and found wide usage [29, 30]. Ortiz et al. screened approximately 600,000 small molecules to assess their growth inhibition effects on* Leishmania* parasites using the SYBR Green I assay [31]. Build on the results from these previous studies, our research extends the application of the SYBR Green I assay to assess drug sensitivity in *T. vaginalis* parasites. The key strategy of this method involves modifying the TYM medium by replacing yeast extract and tryptone with RPMI 1640 and Tryptone Plus, respectively, which minimizes the background florescent signal and prevents fluorescence overlay from the interaction of nucleotide content in the medium with parasite DNA. RPMI 1640, a modified version of McCoy’s 5A medium, is extensively used to support the growth of a wide range of cell lines. In the present study, we confirmed RPMI 1640 as an ideal substitute for yeast extract in the fluorescence assay due to its absence of nucleic content. Tryptone Plus, which is derived from a digest of casein as tryptone, is of a better quality and higher solubility than tryptone, and is more suitable for biotechnological applications according to the manufacturer’s description [32]. Consequently, our modified TSF assay medium shows a similar ability to support parasite growth as the standard MCL method but with significantly reduced background fluorescent signals.

While both the 48-h and 72-h incubations with drugs yielded sigmoid assay curves, the 48-h incubation period exhibited greater sensitivity in determining drug resistance compared to the 72-h period. This observation might be attributable to the growth and continuous replication of a subset of parasites that displays tolerance to antimicrobial drugs during a longer incubation time. It is noteworthy that the 48-h incubation condition is also employed in the standard MLC method, where parasites are treated with drug for 48 h and subsequently inspected under a microscope to detect any viable parasites. In our study, the trends of IC_50_ values for MTZ, TDZ, ODZ and SDZ for the resistant isolate (ATCC 50143) are consistent with the results obtained using the MLC method in the 48-h group, further underscoring the reliability of this incubation condition.

The IC_50_s obtained from the TSF assay were found to be consistent with the results from the standard MLC method for MTZ, TDZ, ODZ and SDZ, suggesting the potential utility of this method for assessing the efficacy of antimicrobial agents against *T. vaginalis *in vitro. Additionally, this method overcomes several limitations associated with the MLC method. One notable disadvantage of the MLC method is that the microscopy-based results might provide inconsistent results, as the accuracy of the values can be influenced by the individuals interpreting the parasite morphology. In contrast, the fluorescence values obtained in the TSF assay remain consistent regardless of the researcher conducting the measurements. Furthermore, the MLC method provides information on drug efficacy within a relatively wide concentration range, as it only determines the MLC from a single well while the IC_50_ values calculated in the TSF assay are derived from a panel of wells and provide relatively sensitive results. The findings of the present study highlight the potential of the TSF assay as a high-throughput method for drug screening and monitoring drug resistance in *T. vaginalis*. However, further evaluation is necessary before this technique can be employed for large-scale screening and resistance supervision.

## Conclusion

The emergence and dissemination of drug resistance in *T. vaginalis* parasites has become a major concern in trichomoniasis treatment, necessitating close supervision. In the present study, we introduce a SYBR Green I-based fluorescence measurement for monitoring drug resistance and screening novel therapeutic agents. Compared to the conventional MLC method, the TSF assay demonstrates superior efficiency and allows a plate reader to generate results, thereby avoiding assessment subjectivity and reducing the time needed for the assay due to manual inspection of live parasites under a microscope, as required by the MLC method. The results of this study indicate the potential utility of the TSF assay in drug screening and suggest its viability as a high-throughput screening assay.

### Supplementary Information

**Additional file 1: Figure S1.** Evaluation of *T. vaginalis* growth under anaerobic or aerobic cultivation condition in TYM or optimized TSF medium. The growth curves were obtained by counting parasite densities every 12 h for three consecutive days.

**Additional file 2: Table S1.** Evaluation of media with different components in support of *T. vaginalis* growth. The parasite densities were counted daily. R1 and R2 represent two biological replicates.

**Additional file 3: Table S2.** The background fluorescent signals of tested media. R represents the average of two technical replicates, while R1, R2 and R3 represent three biological replicates.

**Additional file 4: Table S3.** Evaluation of *T. vaginalis* growth under anaerobic and aerobic cultivation conditions in TYM or optimized TSF medium. Parasites cultivated under different conditions were counted every 12 h. R1 and R2 represent two biological replicates.

**Additional file 5: Table S4.** Assessment of fluorescence linearity. The optical density values measured at different parasite concentrations are presented. R represents the average of two technical replicates, while R1, R2, and R3 represent three biological replicates.

**Additional file 6: Table S5.** The optical density values of the fluorescent signals obtained from comparison experiment of TSF setup concentration and incubation conditions.

**Additional file 7: Table S6.** The optical density values of the fluorescent signals obtained from validation of TSF assay conditions utilizing isolates with various responses against four antimicrobial drugs.

## Data Availability

All data generated or analyzed during this study are included in this published article.

## Abbreviations

EDTA: Ethylene diamine tetraacetic acid
HIV: Human immunodeficiency virus
IC_50_: 50% inhibitory concentration
MLC: Minimum lethal concentration
MTZ: Metronidazole
ODZ: Ornidazole
RPMI: Roswell Park Memorial Institute
SDZ: Secnidazole
STI: Sexually transmitted infection
TDZ: Tinidazole
TSF: *T. vaginalis* SYBR Green I-based fluorescence
TV: *Trichomonas vaginalis*
TYM: Trypticase-yeast extract-maltose
