# Supplementary material for: A fluorescence-based assay for Trichomonas vaginalis drug screening
Source: Parasit Vectors. 2023 Sep 18;16:329. doi: 10.1186/s13071-023-05919-6 (PMC10507874; doi:10.1186/s13071-023-05919-6)
Supplement: Supplementary file 1 — Additional file 1: Figure S1. Evaluation of T. vaginalis growth under anaerobic or aerobic cultivation condition in TYM or optimized TSF medium. The growth curves were obtained by counting parasite densities every 12 h for three consecutive days. [file 13071_2023_5919_MOESM1_ESM.docx]

Additional File

**A Fluorescence-Based Assay** **for *Trichomonas vaginalis* Drug Screening**

Qianqian Chen^1†^, Jingzhong Li^2†^, Zhensheng Wang^3^, Wei Meng^1^, Heng Wang^3^, Zenglei Wang^1*^


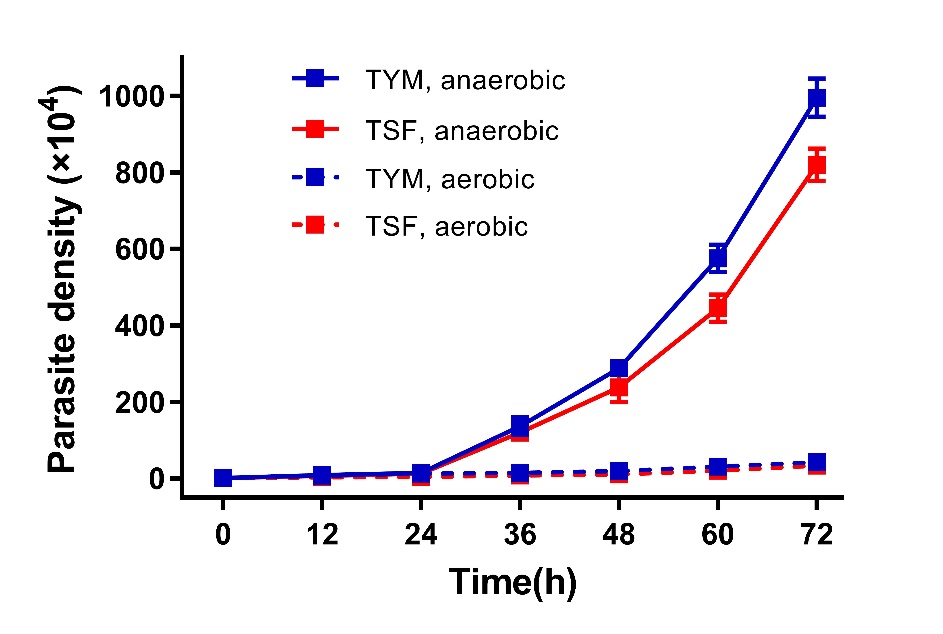


**Fig. S1.** Evaluation of *T. vaginalis* growth under anaerobic or aerobic cultivation condition in TYM or optimized TSF medium. The growth curves were obtained by counting parasite densities every 12 hours for three consecutive days.
