# Supplementary material for: A fluorescence-based assay for Trichomonas vaginalis drug screening
Source: Parasit Vectors. 2023 Sep 18;16:329. doi: 10.1186/s13071-023-05919-6 (PMC10507874; doi:10.1186/s13071-023-05919-6)
Supplement: Supplementary file 2 — Additional file 2: Table S1. Evaluation of media with different components in support of T. vaginalis growth. The parasite densities were counted daily. R1 and R2 represent two biological replicates. [file 13071_2023_5919_MOESM2_ESM.docx]

Additional File

**A Fluorescence-Based Assay** **for *Trichomonas vaginalis* Drug Screening**

Qianqian Chen^1†^, Jingzhong Li^2†^, Zhensheng Wang^3^, Wei Meng^1^, Heng Wang^3^, Zenglei Wang^1*^

**Table S1.** Evaluation of media with different components in support of *T. vaginalis* growth. The parasite densities were counted daily. R1 and R2 represent two biological replicates.

| Days | Parasite density (×10^4^ parasite per ml) | | | | | | | | | | | |
| --- | --- | --- | --- | --- | --- | --- | --- | --- | --- | --- | --- | --- |
|  | Test 1 | | Test 2 | | Test 3 | | Test 4 | | Test 5 | | TYM | |
|  | R1 | R2 | R1 | R2 | R1 | R2 | R1 | R2 | R1 | R2 | R1 | R2 |
| 1 | 0.7 | 1.2 | 1 | 1 | 1.5 | 0.7 | 0.5 | 0.6 | 1.7 | 1.5 | 1 | 0.8 |
| 2 | 18 | 17 | 29 | 35 | 46 | 32 | 10 | 13 | 25 | 19 | 71 | 53 |
| 3 | 55 | 43 | 171 | 199 | 210 | 186 | 80 | 76 | 196 | 220 | 242 | 198 |
| 4 | 312 | 350 | 412 | 341 | 483 | 575 | 362 | 391 | 1030 | 787 | 587 | 750 |
| 5 | 1.5 | 0.9 | 0.7 | 0.6 | 1.7 | 0.9 | 1.9 | 0.7 | 0.8 | 1 | 1.2 | 1.1 |
| 6 | 38 | 43 | 43 | 60 | 204 | 168 | 86 | 105 | 312 | 250 | 350 | 241 |
| 7 | 175 | 266 | 350 | 300 | 512 | 458 | 450 | 416 | 762 | 700 | 887 | 666 |
