# Supplementary material for: A fluorescence-based assay for Trichomonas vaginalis drug screening
Source: Parasit Vectors. 2023 Sep 18;16:329. doi: 10.1186/s13071-023-05919-6 (PMC10507874; doi:10.1186/s13071-023-05919-6)
Supplement: Supplementary file 3 — Additional file 3: Table S2. The background fluorescent signals of tested media. R represents the average of two technical replicates, while R1, R2 and R3 represent three biological replicates. [file 13071_2023_5919_MOESM3_ESM.docx]

Additional File

**A Fluorescence-Based Assay** **for *Trichomonas vaginalis* Drug Screening**

Qianqian Chen^1†^, Jingzhong Li^2†^, Zhensheng Wang^3^, Wei Meng^1^, Heng Wang^3^, Zenglei Wang^1*^

**Table S2.** The background fluorescence signals of tested media. R represents the average of two technical replicates, while R1, R2, and R3 represent three biological replicates.

| Group | Optical density values | | |
| --- | --- | --- | --- |
|  | R1 | R2 | R3 |
| Test 1 | 9889 | 9339 | 9503 |
| Test 2 | 14662 | 14153 | 13810 |
| Test 3 | 7221 | 7037 | 6803 |
| Test 4 | 13798 | 13396 | 13651 |
| Test 5 | 9409 | 9479 | 9422 |
| TYM | 35831 | 34722 | 33976 |
