# Supplementary material for: A fluorescence-based assay for Trichomonas vaginalis drug screening
Source: Parasit Vectors. 2023 Sep 18;16:329. doi: 10.1186/s13071-023-05919-6 (PMC10507874; doi:10.1186/s13071-023-05919-6)
Supplement: Supplementary file 4 — Additional file 4: Table S3. Evaluation of T. vaginalis growth under anaerobic and aerobic cultivation conditions in TYM or optimized TSF medium. Parasites cultivated under different conditions were counted every 12 h. R1 and R2 represent two biological replicates. [file 13071_2023_5919_MOESM4_ESM.docx]

Additional File

**A Fluorescence-Based Assay** **for *Trichomonas vaginalis* Drug Screening**

Qianqian Chen^1†^, Jingzhong Li^2†^, Zhensheng Wang^3^, Wei Meng^1^, Heng Wang^3^, Zenglei Wang^1*^

**Table S3.** Evaluation of *T. vaginalis* growth under anaerobic and aerobic cultivation conditions in TYM or optimized TSF medium. Parasites cultivated under different conditions was counted every 12 hours. R1 and R2 represent two biological replicates.

| Incubation time (h) | Parasite density (×10^4^ parasite per ml) | | | | | | | |
| --- | --- | --- | --- | --- | --- | --- | --- | --- |
|  | Aerobic condition | | | | Anaerobic condition | | | |
|  | TYM medium | | TSF medium | | TYM medium | | TSF medium | |
|  | R1 | R2 | R1 | R2 | R1 | R2 | R1 | R2 |
| 0 | 1 | 1 | 1 | 1 | 1 | 1 | 1 | 1 |
| 12 | 5 | 9 | 6 | 1 | 16 | 2 | 12 | 2 |
| 24 | 15 | 11 | 6 | 3 | 18 | 11 | 14 | 11 |
| 36 | 17 | 12 | 9 | 7 | 120 | 153 | 116 | 125 |
| 48 | 16 | 23 | 10 | 12 | 275 | 300 | 265 | 210 |
| 60 | 24 | 38 | 19 | 22 | 600 | 550 | 420 | 470 |
| 72 | 44 | 41 | 33 | 35 | 960 | 1030 | 850 | 790 |
