# Supplementary material for: A fluorescence-based assay for Trichomonas vaginalis drug screening
Source: Parasit Vectors. 2023 Sep 18;16:329. doi: 10.1186/s13071-023-05919-6 (PMC10507874; doi:10.1186/s13071-023-05919-6)
Supplement: Supplementary file 5 — Additional file 5: Table S4. Assessment of fluorescence linearity. The optical density values measured at different parasite concentrations are presented. R represents the average of two technical replicates, while R1, R2, and R3 represent three biological replicates. [file 13071_2023_5919_MOESM5_ESM.docx]

Additional File

**A Fluorescence-Based Assay** **for *Trichomonas vaginalis* Drug Screening**

Qianqian Chen^1†^, Jingzhong Li^2†^, Zhensheng Wang^3^, Wei Meng^1^, Heng Wang^3^, Zenglei Wang^1*^

**Table S4.** Assessment of fluorescence linearity. The Optical density values measured at different parasites concentrations were presented R represents the average of two technical replicates, while R1, R2, and R3 represent three biological replicates.

| Parasites density (×10^6^ parasites per ml) | Optical density values | | |
| --- | --- | --- | --- |
|  | R1 | R2 | R3 |
| 10 | 67678 | 67334 | 69269 |
| 5 | 43455 | 42652 | 44237 |
| 2.5 | 28370 | 27295 | 28055 |
| 1.25 | 20344 | 19955 | 20134 |
| 0.625 | 16875 | 15309 | 16101 |
| 0.313 | 14861 | 14538 | 14622 |
| 0.156 | 14015 | 13962 | 13994 |
| 0.078 | 13715 | 13796 | 13438 |
| 0.039 | 13467 | 13330 | 13013 |
| 0 | 10238 | 10029 | 10081 |
