# Supplementary material for: A fluorescence-based assay for Trichomonas vaginalis drug screening
Source: Parasit Vectors. 2023 Sep 18;16:329. doi: 10.1186/s13071-023-05919-6 (PMC10507874; doi:10.1186/s13071-023-05919-6)
Supplement: Supplementary file 6 — Additional file 6: Table S5. The optical density values of the fluorescent signals obtained from comparison experiment of TSF setup concentration and incubation conditions. [file 13071_2023_5919_MOESM6_ESM.docx]

Additional File

**A Fluorescence-Based Assay** **for *Trichomonas vaginalis* Drug Screening**

Qianqian Chen^1†^, Jingzhong Li^2†^, Zhensheng Wang^3^, Wei Meng^1^, Heng Wang^3^, Zenglei Wang^1*^

**Table S5.** The fluorescent optical density values obtained from comparison experiment of TSF setup concentration and incubation conditions. Table **S5-1** demonstrates the optical density values from tests with the incubation time of 24 hours, **S5-2** presents the data from tests with the incubation time of 48 hours, and **S5-3** shows data from tests with the incubation time of 72 hours. R1, R2, and R3 represent three biological replicates.

**Table S5-1.**

| Drug  concentrations  (μg/ml) | Optical density values | | | | | | | | | | | | |
| --- | --- | --- | --- | --- | --- | --- | --- | --- | --- | --- | --- | --- | --- |
|  | 6×10^4^/ml | | | 3×10^4^/ml | | | 1.5×10^4^/ml | | | | 1×10^4^/ml | | |
|  | R1 | R2 | R3 | R1 | R2 | R3 | R1 | R2 | R3 | R1 | | R2 | R3 |
| 1600 | 12582 | 13937 | 13446 | 14241 | 15317 | 14836 | 14897 | 16138 | 15959 | 15204 | | 16726 | 16413 |
| 800 | 14860 | 15639 | 15194 | 15970 | 17051 | 16841 | 16691 | 17887 | 17699 | 17235 | | 18645 | 18516 |
| 400 | 15695 | 16743 | 16318 | 16244 | 17918 | 17826 | 17972 | 18603 | 19242 | 19022 | | 19719 | 19667 |
| 200 | 16167 | 16995 | 17054 | 16984 | 18483 | 18737 | 18879 | 19504 | 19413 | 19585 | | 19911 | 20187 |
| 100 | 16301 | 17272 | 17203 | 17357 | 18258 | 18820 | 19222 | 19469 | 19193 | 20040 | | 20554 | 21082 |
| 50 | 17054 | 18256 | 17884 | 18168 | 19158 | 19312 | 19089 | 19938 | 19738 | 20226 | | 20801 | 20786 |
| 25 | 19001 | 19584 | 19369 | 17994 | 19302 | 19710 | 19914 | 19799 | 20692 | 20544 | | 20617 | 20640 |
| 12.5 | 19448 | 21426 | 21170 | 18792 | 20274 | 20524 | 20537 | 20834 | 21226 | 20687 | | 21367 | 21780 |
| 6.25 | 21628 | 22263 | 22913 | 19385 | 20353 | 21050 | 20221 | 21371 | 20907 | 20693 | | 21847 | 21221 |
| 3.125 | 22488 | 22399 | 22962 | 19255 | 20361 | 20399 | 20831 | 21573 | 21055 | 21058 | | 21873 | 21388 |
| 1.563 | 21750 | 22917 | 23870 | 19873 | 20671 | 20742 | 20169 | 20710 | 21415 | 20560 | | 21706 | 20999 |
| 0.781 | 22344 | 23943 | 24848 | 20103 | 21440 | 20660 | 20181 | 21498 | 20805 | 20853 | | 21737 | 20809 |
| 0.391 | 22843 | 23042 | 23569 | 20419 | 21441 | 20398 | 20649 | 21777 | 20644 | 21017 | | 22062 | 21490 |
| 0.195 | 22397 | 23390 | 22302 | 19904 | 21532 | 20246 | 20345 | 21623 | 20006 | 20959 | | 22221 | 21014 |
| 0.098 | 22276 | 23729 | 23651 | 19817 | 21330 | 20128 | 20162 | 21277 | 20638 | 21238 | | 22008 | 21459 |
| 0.049 | 22532 | 23451 | 24144 | 19541 | 20918 | 19758 | 20081 | 20377 | 20508 | 21274 | | 22212 | 21389 |
| 0.024 | 22880 | 22840 | 24495 | 19866 | 21220 | 20231 | 20351 | 21097 | 20222 | 19954 | | 22022 | 21365 |
| 0.012 | 22329 | 23292 | 25056 | 19763 | 20995 | 20440 | 20785 | 21237 | 20887 | 20966 | | 22096 | 21706 |
| 0.006 | 22889 | 23515 | 25060 | 20283 | 20781 | 20818 | 20630 | 21212 | 21533 | 21012 | | 21337 | 22029 |
| 0 | 23358 | 23048 | 24337 | 20267 | 20686 | 21657 | 20470 | 21592 | 22040 | 20882 | | 21711 | 21664 |

**Table S5-2.**

| Drug  concentrations  (μg/ml) | Optical density values | | | | | | | | | | | | |
| --- | --- | --- | --- | --- | --- | --- | --- | --- | --- | --- | --- | --- | --- |
|  | 6×10^4^/ml | | | 3×10^4^/ml | | | 1.5×10^4^/ml | | | | 1×10^4^/ml | | |
|  | R1 | R2 | R3 | R1 | R2 | R3 | R1 | R2 | R3 | R1 | | R2 | R3 |
| 1600 | 5440 | 4795 | 4878 | 5667 | 5041 | 5167 | 5602 | 4813 | 4999 | 5772 | | 5054 | 5166 |
| 800 | 6428 | 5552 | 5874 | 6423 | 5712 | 6022 | 6047 | 5487 | 5779 | 6525 | | 5951 | 6056 |
| 400 | 6370 | 6117 | 6135 | 6900 | 6358 | 6684 | 6522 | 6157 | 6610 | 7181 | | 6573 | 6735 |
| 200 | 6960 | 6409 | 6391 | 7277 | 6676 | 7037 | 6964 | 6564 | 6973 | 7572 | | 7066 | 7370 |
| 100 | 7243 | 6617 | 6707 | 7403 | 6627 | 7222 | 7343 | 7019 | 7108 | 8058 | | 7420 | 7627 |
| 50 | 8399 | 7738 | 6784 | 7393 | 6803 | 6954 | 7585 | 6850 | 7177 | 8121 | | 7278 | 7611 |
| 25 | 12372 | 11950 | 10235 | 10981 | 8945 | 10034 | 8497 | 7771 | 7950 | 8377 | | 7548 | 7955 |
| 12.5 | 19233 | 17091 | 15651 | 14883 | 12756 | 13408 | 12675 | 11532 | 12482 | 10281 | | 9177 | 9834 |
| 6.25 | 30694 | 24628 | 23927 | 19879 | 17093 | 17479 | 16137 | 14179 | 14725 | 14982 | | 13304 | 14189 |
| 3.125 | 34546 | 30206 | 31508 | 27593 | 25853 | 26364 | 19878 | 17650 | 18703 | 17137 | | 14159 | 16159 |
| 1.563 | 29696 | 32605 | 37580 | 32927 | 31304 | 35727 | 21055 | 19522 | 23899 | 18149 | | 16147 | 17857 |
| 0.781 | 30971 | 31479 | 33557 | 35606 | 35076 | 35679 | 24343 | 25381 | 27412 | 20694 | | 19593 | 21347 |
| 0.391 | 32718 | 32828 | 35953 | 36820 | 36089 | 41693 | 24510 | 27226 | 28745 | 20843 | | 20066 | 21694 |
| 0.195 | 31597 | 34561 | 40442 | 38565 | 38957 | 40281 | 28155 | 26899 | 30020 | 20673 | | 19464 | 24029 |
| 0.098 | 32116 | 34885 | 37265 | 37061 | 36985 | 42744 | 29882 | 27979 | 29323 | 23313 | | 22062 | 25061 |
| 0.049 | 33368 | 33624 | 38345 | 40074 | 38303 | 43401 | 28727 | 25301 | 27549 | 22350 | | 20578 | 22599 |
| 0.024 | 35372 | 33154 | 38693 | 40511 | 37887 | 39705 | 28340 | 27229 | 27710 | 24029 | | 19524 | 21807 |
| 0.012 | 35889 | 34884 | 37844 | 40190 | 37885 | 42673 | 27885 | 28488 | 28759 | 23329 | | 22229 | 22198 |
| 0.006 | 34098 | 36778 | 36753 | 39920 | 38660 | 39469 | 27733 | 26355 | 28737 | 22460 | | 20502 | 21628 |
| 0 | 37381 | 36118 | 43566 | 39694 | 37642 | 40418 | 26693 | 25773 | 26010 | 20997 | | 19324 | 22163 |

**Table S5-3.**

| Drug  concentrations  (μg/ml) | Optical density values | | | | | | | | | | | | |
| --- | --- | --- | --- | --- | --- | --- | --- | --- | --- | --- | --- | --- | --- |
|  | 6×10^4^/ml | | | 3×10^4^/ml | | | 1.5×10^4^/ml | | | | 1×10^4^/ml | | |
|  | R1 | R2 | R3 | R1 | R2 | R3 | R1 | R2 | R3 | R1 | | R2 | R3 |
| 1600 | 4881 | 4716 | 4898 | 4862 | 4941 | 4899 | 4719 | 4494 | 4718 | 4742 | | 4570 | 4695 |
| 800 | 5599 | 5639 | 5779 | 5857 | 5692 | 5856 | 5667 | 5472 | 5550 | 5861 | | 5531 | 5815 |
| 400 | 6602 | 6114 | 6476 | 6531 | 6297 | 6477 | 6385 | 6210 | 6012 | 6490 | | 6184 | 6439 |
| 200 | 6775 | 6814 | 6977 | 7043 | 6907 | 6858 | 6832 | 6618 | 6592 | 7022 | | 6923 | 7027 |
| 100 | 7076 | 6993 | 7162 | 7231 | 7120 | 7226 | 7027 | 6920 | 6829 | 7257 | | 7286 | 7444 |
| 50 | 7336 | 7514 | 7854 | 7464 | 7398 | 7283 | 7246 | 7086 | 7067 | 7785 | | 7717 | 7499 |
| 25 | 8694 | 9833 | 9939 | 8337 | 9539 | 9022 | 7534 | 7859 | 7385 | 7936 | | 7534 | 7904 |
| 12.5 | 13701 | 16658 | 20100 | 11876 | 14100 | 12729 | 11802 | 11626 | 11013 | 10248 | | 11690 | 12115 |
| 6.25 | 24755 | 22721 | 24734 | 17226 | 24436 | 22922 | 16796 | 16626 | 13415 | 13969 | | 15798 | 17882 |
| 3.125 | 25980 | 24901 | 27649 | 28612 | 23891 | 24308 | 28051 | 28414 | 24862 | 23196 | | 25478 | 26502 |
| 1.563 | 27267 | 29638 | 28984 | 24936 | 31722 | 32739 | 28989 | 28143 | 30784 | 33369 | | 34393 | 31530 |
| 0.781 | 29515 | 33779 | 38669 | 28057 | 36539 | 40526 | 31223 | 31046 | 30821 | 31388 | | 32861 | 34646 |
| 0.391 | 29945 | 37594 | 33302 | 32460 | 36353 | 42948 | 34268 | 35453 | 44004 | 30255 | | 35323 | 44082 |
| 0.195 | 30348 | 39571 | 42657 | 29277 | 39712 | 46241 | 35491 | 35041 | 45770 | 33684 | | 36268 | 43844 |
| 0.098 | 30644 | 38326 | 45333 | 31474 | 37563 | 45383 | 41775 | 37659 | 42895 | 32378 | | 38568 | 44800 |
| 0.049 | 31351 | 37562 | 44763 | 32135 | 34389 | 38105 | 40375 | 34764 | 40810 | 34319 | | 36629 | 42225 |
| 0.024 | 31765 | 39349 | 40147 | 36019 | 37943 | 46107 | 42511 | 39893 | 43012 | 35196 | | 38988 | 42469 |
| 0.012 | 34123 | 38330 | 41320 | 33849 | 38845 | 45107 | 39636 | 40591 | 47032 | 37177 | | 38772 | 44820 |
| 0.006 | 36446 | 36704 | 39249 | 33260 | 37378 | 45930 | 34544 | 39054 | 41569 | 35055 | | 36733 | 41961 |
| 0 | 38689 | 37581 | 40163 | 35508 | 36776 | 44362 | 35873 | 38543 | 37111 | 36795 | | 38090 | 40388 |
