# Supplementary material for: A fluorescence-based assay for Trichomonas vaginalis drug screening
Source: Parasit Vectors. 2023 Sep 18;16:329. doi: 10.1186/s13071-023-05919-6 (PMC10507874; doi:10.1186/s13071-023-05919-6)
Supplement: Supplementary file 7 — Additional file 7: Table S6. The optical density values of the fluorescent signals obtained from validation of TSF assay conditions utilizing isolates with various responses against four antimicrobial drugs. [file 13071_2023_5919_MOESM7_ESM.docx]

Additional File

**A Fluorescence-Based Assay** **for *Trichomonas vaginalis* Drug Screening**

Qianqian Chen^1†^, Jingzhong Li^2†^, Zhensheng Wang^3^, Wei Meng^1^, Heng Wang^3^, Zenglei Wang^1*^

**Table S6.** The fluorescent optical density values obtained from validation of TSF assay conditions utilizing isolates with various responses against four antimicrobial drugs. Table **S6-1** presents the optical density values from the experiments using TV-315 isolate against four antimicrobial drugs at a setup concentration of 3×10^4^ parasites per ml and an incubation time of 48 hour. Table **S6-2** indicates the optical density values from the experiments using TV-315 against the drugs at a setup concentration of 1×10^4^/ml and an incubation time of 72 hour. Table **S6-3** shows the data from the experiments using TV-334 against the drugs at an initial parasite concentration of 3×10^4^/ml and an incubation time of 48 hour. Table **S6-4** demonstrates the data from the experiment using TV-334 against four drugs at an initial parasite concentration of 1×10^4^/ml and an incubation time of 72 hour. Table **S6-5** displays the data from the experiment using TV-689 against four drugs at an initial concentration of 3×10^4^ parasites per ml and an incubation time of 48 hour. Table **S6-6** shows data from the experiment using TV-689 against four drugs at an initial parasite concentration of 1×10^4^/ml and an incubation time of 72 hour. Table **S6-7** demonstrates data from the experiment using ATCC50143 against four drugs at an initial parasite concentration of 3×10^4^/ml and an incubation time of 48 hour. Table **S6-8** presents data from the experiment using ATCC50143 against four drugs at an initial parasite concentration of 1×10^4^/ml and an incubation time of 72 hour. R1, R2, and R3 represent three biological replicates.

**Table S6-1.**

| Drug  concentrations  (μg/ml) | Optical density value | | | | | | | | | | | | |
| --- | --- | --- | --- | --- | --- | --- | --- | --- | --- | --- | --- | --- | --- |
|  | MTZ | | | TDZ | | | ODZ | | | | SDZ | | |
|  | R1 | R2 | R3 | R1 | R2 | R3 | R1 | R2 | R3 | R1 | | R2 | R3 |
| 1600 | 5065 | 4943 | 3881 | 4093 | 3343 | 3559 | 5061 | 4961 | 4423 | 4351 | | 3910 | 4222 |
| 800 | 5811 | 5894 | 5027 | 4844 | 4030 | 4594 | 5656 | 5617 | 4994 | 4917 | | 4683 | 4990 |
| 400 | 6460 | 6422 | 5740 | 5356 | 4575 | 5148 | 6179 | 6080 | 5947 | 5537 | | 5302 | 5627 |
| 200 | 6990 | 6795 | 5951 | 5513 | 5109 | 5512 | 6757 | 6659 | 6309 | 5893 | | 5681 | 6056 |
| 100 | 7431 | 7555 | 6932 | 5608 | 5504 | 5866 | 7302 | 6990 | 6791 | 6502 | | 6494 | 6889 |
| 50 | 7909 | 7938 | 7608 | 5564 | 5758 | 6090 | 7752 | 7530 | 7235 | 7212 | | 7583 | 7953 |
| 25 | 10422 | 10540 | 10155 | 5842 | 6253 | 6417 | 8406 | 7953 | 7919 | 10720 | | 11227 | 10959 |
| 12.5 | 14981 | 14840 | 14676 | 6274 | 7142 | 6901 | 9795 | 9090 | 9551 | 14312 | | 15736 | 15102 |
| 6.25 | 19280 | 18591 | 18610 | 6888 | 8087 | 7768 | 14886 | 13251 | 14472 | 18605 | | 19867 | 19528 |
| 3.125 | 24893 | 22734 | 23875 | 10907 | 12124 | 11938 | 22492 | 20019 | 21927 | 21948 | | 25043 | 24470 |
| 1.563 | 29603 | 30252 | 25848 | 25242 | 26412 | 25394 | 35567 | 33615 | 34086 | 40331 | | 37372 | 37222 |
| 0.781 | 37800 | 39571 | 33382 | 38244 | 37174 | 41808 | 45558 | 44603 | 42655 | 46814 | | 46572 | 44084 |
| 0.391 | 45031 | 47343 | 42776 | 44339 | 37906 | 39590 | 51974 | 52185 | 49428 | 50510 | | 49406 | 49977 |
| 0.195 | 51021 | 51019 | 47182 | 43988 | 39510 | 43770 | 55514 | 52549 | 51830 | 52490 | | 50028 | 50293 |
| 0.098 | 52640 | 52480 | 49250 | 45149 | 42329 | 43543 | 56391 | 49417 | 47525 | 48353 | | 50282 | 52340 |
| 0.049 | 52641 | 51414 | 50820 | 43925 | 42896 | 41734 | 57425 | 51534 | 49609 | 47877 | | 50188 | 52375 |
| 0.024 | 52518 | 52846 | 49891 | 41632 | 43882 | 41651 | 56503 | 52346 | 51135 | 47152 | | 51793 | 53276 |
| 0.012 | 51848 | 51567 | 51599 | 42116 | 46123 | 42346 | 56334 | 53018 | 52484 | 50145 | | 50425 | 51098 |
| 0.006 | 54339 | 51101 | 49380 | 39741 | 46558 | 42706 | 55640 | 53084 | 54020 | 49008 | | 53350 | 51299 |
| 0 | 53794 | 46574 | 53446 | 38073 | 46106 | 41745 | 53519 | 52527 | 54333 | 50301 | | 50643 | 50358 |

**Table S6-2.**

| Drug  concentrations  (μg/ml) | Optical density value | | | | | | | | | | | | |
| --- | --- | --- | --- | --- | --- | --- | --- | --- | --- | --- | --- | --- | --- |
|  | MTZ | | | TDZ | | | ODZ | | | | SDZ | | |
|  | R1 | R2 | R3 | R1 | R2 | R3 | R1 | R2 | R3 | R1 | | R2 | R3 |
| 1600 | 4637 | 4633 | 4302 | 4158 | 4002 | 4246 | 4226 | 4454 | 3943 | 4061 | | 3847 | 4125 |
| 800 | 5278 | 5288 | 4806 | 4703 | 4646 | 4948 | 4860 | 4827 | 4398 | 4518 | | 4477 | 4747 |
| 400 | 5716 | 5800 | 5332 | 5278 | 4918 | 5279 | 5307 | 5426 | 4940 | 5040 | | 4858 | 5336 |
| 200 | 6162 | 6142 | 5812 | 5551 | 5676 | 5949 | 5936 | 5949 | 5523 | 5551 | | 5438 | 5915 |
| 100 | 6666 | 6613 | 6256 | 5898 | 6305 | 6454 | 6311 | 6310 | 6053 | 5654 | | 6062 | 6326 |
| 50 | 7280 | 7080 | 6927 | 5964 | 6453 | 6535 | 6730 | 6610 | 6450 | 6288 | | 6576 | 6977 |
| 25 | 6970 | 7858 | 7850 | 6244 | 6666 | 6920 | 7089 | 6828 | 6863 | 7374 | | 7539 | 7673 |
| 12.5 | 11164 | 11511 | 12767 | 6349 | 6983 | 7261 | 7303 | 6981 | 7221 | 11900 | | 12957 | 12396 |
| 6.25 | 15659 | 16654 | 17584 | 6825 | 7408 | 7221 | 8584 | 8463 | 9089 | 16166 | | 18330 | 18087 |
| 3.125 | 25154 | 24429 | 29329 | 7479 | 8230 | 7446 | 15368 | 15102 | 16470 | 24764 | | 26369 | 25628 |
| 1.563 | 35554 | 35654 | 33021 | 18505 | 18605 | 12998 | 28069 | 28119 | 25636 | 31459 | | 30128 | 31351 |
| 0.781 | 39665 | 39156 | 35598 | 41558 | 39030 | 39464 | 36443 | 40780 | 33056 | 32327 | | 29613 | 36492 |
| 0.391 | 41237 | 39280 | 38973 | 39573 | 40460 | 40499 | 38250 | 41168 | 36440 | 36695 | | 31903 | 34135 |
| 0.195 | 41722 | 42777 | 44539 | 43305 | 45579 | 39716 | 36408 | 44825 | 36172 | 42175 | | 37594 | 35089 |
| 0.098 | 45676 | 45987 | 44571 | 49115 | 45479 | 40755 | 40021 | 43459 | 36820 | 40605 | | 35700 | 34993 |
| 0.049 | 39280 | 48405 | 44972 | 50623 | 46942 | 44550 | 38749 | 40842 | 38531 | 36680 | | 42964 | 34745 |
| 0.024 | 41777 | 52098 | 46927 | 49240 | 48719 | 45313 | 40195 | 43096 | 41184 | 36666 | | 39931 | 34771 |
| 0.012 | 45108 | 48699 | 43997 | 45662 | 45705 | 43396 | 40088 | 41049 | 46131 | 38579 | | 36604 | 37185 |
| 0.006 | 47225 | 49349 | 42342 | 43760 | 43358 | 39318 | 40694 | 38953 | 44380 | 34985 | | 38256 | 34848 |
| 0 | 45624 | 50359 | 44190 | 40889 | 44450 | 44598 | 39757 | 41028 | 39556 | 34283 | | 39349 | 36265 |

**Table S6-3.**

| Drug  concentrations  (μg/ml) | Optical density value | | | | | | | | | | | | |
| --- | --- | --- | --- | --- | --- | --- | --- | --- | --- | --- | --- | --- | --- |
|  | MTZ | | | TDZ | | | ODZ | | | | SDZ | | |
|  | R1 | R2 | R3 | R1 | R2 | R3 | R1 | R2 | R3 | R1 | | R2 | R3 |
| 1600 | 6221 | 6201 | 6048 | 5753 | 5876 | 5883 | 6349 | 6392 | 6305 | 6109 | | 6272 | 6209 |
| 800 | 7335 | 7374 | 6980 | 7328 | 7285 | 7623 | 7336 | 7173 | 6874 | 6623 | | 6803 | 7079 |
| 400 | 7963 | 7840 | 7625 | 8229 | 8123 | 8688 | 8057 | 7687 | 7523 | 7364 | | 7441 | 7680 |
| 200 | 8682 | 8495 | 8270 | 8777 | 8747 | 9305 | 8749 | 8521 | 8400 | 8158 | | 8037 | 8705 |
| 100 | 9102 | 8704 | 8418 | 8867 | 9108 | 9859 | 9623 | 8865 | 8934 | 8557 | | 8512 | 9108 |
| 50 | 9565 | 8786 | 8765 | 8970 | 9311 | 10110 | 9718 | 9450 | 9396 | 8760 | | 9062 | 9853 |
| 25 | 10560 | 9860 | 10182 | 9213 | 9775 | 10211 | 10006 | 9613 | 9556 | 10382 | | 10680 | 10669 |
| 12.5 | 12375 | 11823 | 12587 | 9464 | 9934 | 10165 | 10325 | 9885 | 10292 | 13443 | | 13769 | 14627 |
| 6.25 | 17578 | 17096 | 17675 | 9402 | 10054 | 10489 | 12283 | 11955 | 11678 | 19540 | | 19148 | 20593 |
| 3.125 | 25865 | 23732 | 26262 | 11079 | 11381 | 11846 | 19066 | 18493 | 17860 | 29755 | | 28855 | 29786 |
| 1.563 | 31518 | 30930 | 31357 | 22074 | 19791 | 18364 | 28959 | 27614 | 24716 | 31574 | | 30240 | 32655 |
| 0.781 | 38654 | 35982 | 36435 | 31934 | 32648 | 31470 | 30572 | 30149 | 30105 | 35191 | | 34926 | 35822 |
| 0.391 | 39020 | 38203 | 37678 | 38390 | 35151 | 38959 | 34322 | 33153 | 32931 | 36824 | | 36852 | 37270 |
| 0.195 | 41299 | 43049 | 39001 | 40116 | 40085 | 38470 | 37341 | 34001 | 34403 | 37975 | | 38058 | 37022 |
| 0.098 | 42472 | 40229 | 39572 | 38894 | 37979 | 41209 | 37469 | 33984 | 40024 | 36018 | | 37301 | 39417 |
| 0.049 | 39735 | 39093 | 42434 | 38752 | 41228 | 41503 | 35757 | 34797 | 42344 | 33403 | | 38491 | 40236 |
| 0.024 | 41353 | 41923 | 38390 | 40608 | 38280 | 41461 | 36799 | 35506 | 39112 | 35086 | | 38437 | 38255 |
| 0.012 | 43131 | 42399 | 41618 | 43556 | 42500 | 42312 | 36508 | 35733 | 36395 | 36669 | | 37823 | 40377 |
| 0.006 | 42677 | 40615 | 37309 | 40585 | 42281 | 43519 | 36340 | 33667 | 35625 | 35672 | | 37019 | 36951 |
| 0 | 43064 | 41807 | 42505 | 44067 | 44832 | 43580 | 36946 | 37739 | 40830 | 35167 | | 40014 | 39687 |

**Table S6-4.**

| Drug  concentrations  (μg/ml) | Optical density value | | | | | | | | | | | | |
| --- | --- | --- | --- | --- | --- | --- | --- | --- | --- | --- | --- | --- | --- |
|  | MTZ | | | TDZ | | | ODZ | | | | SDZ | | |
|  | R1 | R2 | R3 | R1 | R2 | R3 | R1 | R2 | R3 | R1 | | R2 | R3 |
| 1600 | 5287 | 5283 | 5183 | 4848 | 4692 | 4834 | 5251 | 5109 | 4848 | 5017 | | 4864 | 5011 |
| 800 | 5897 | 5804 | 5438 | 6157 | 5833 | 6212 | 5791 | 5573 | 5214 | 5234 | | 5204 | 5330 |
| 400 | 6948 | 6497 | 6271 | 6664 | 6664 | 6793 | 6278 | 6044 | 5696 | 5845 | | 5842 | 5922 |
| 200 | 7587 | 7184 | 6846 | 6920 | 7096 | 7479 | 7108 | 6798 | 6356 | 6595 | | 6521 | 6863 |
| 100 | 8204 | 8057 | 7654 | 7326 | 7360 | 7948 | 7750 | 7347 | 6937 | 7283 | | 7424 | 7852 |
| 50 | 8567 | 8087 | 7874 | 7438 | 7583 | 8218 | 8360 | 7682 | 7494 | 7461 | | 7761 | 8299 |
| 25 | 9179 | 9994 | 10138 | 7934 | 8069 | 8556 | 8769 | 7988 | 8246 | 10237 | | 10683 | 9464 |
| 12.5 | 13017 | 14819 | 14116 | 8566 | 8807 | 9277 | 8714 | 8342 | 8323 | 15188 | | 15455 | 14635 |
| 6.25 | 19069 | 21721 | 20810 | 8511 | 9082 | 9125 | 11420 | 11678 | 10578 | 23236 | | 24178 | 21992 |
| 3.125 | 25914 | 28972 | 29107 | 8862 | 9162 | 9171 | 21960 | 21444 | 20455 | 29109 | | 29726 | 30031 |
| 1.563 | 35336 | 34544 | 35037 | 19831 | 12133 | 15074 | 32498 | 32160 | 29299 | 32479 | | 30805 | 28883 |
| 0.781 | 34938 | 36381 | 41116 | 38238 | 36152 | 34740 | 33846 | 35062 | 30068 | 31410 | | 35474 | 38679 |
| 0.391 | 37062 | 42531 | 44712 | 42708 | 39213 | 42047 | 36821 | 35885 | 31214 | 34470 | | 35745 | 38184 |
| 0.195 | 37513 | 37768 | 41104 | 42274 | 44274 | 42568 | 38925 | 36626 | 31826 | 35343 | | 37879 | 37183 |
| 0.098 | 38573 | 40188 | 41198 | 49222 | 51357 | 45855 | 36653 | 34895 | 30677 | 34526 | | 38741 | 36137 |
| 0.049 | 38153 | 40800 | 48920 | 52936 | 51994 | 42544 | 41165 | 37256 | 30667 | 36684 | | 39076 | 34668 |
| 0.024 | 37005 | 47072 | 47431 | 49523 | 47403 | 47631 | 42900 | 38201 | 37266 | 34455 | | 41245 | 40712 |
| 0.012 | 40240 | 44938 | 46488 | 46058 | 50862 | 49055 | 41669 | 39220 | 38812 | 36735 | | 39985 | 38543 |
| 0.006 | 41609 | 50497 | 53040 | 50472 | 44711 | 47302 | 43690 | 34655 | 41255 | 33748 | | 42755 | 38084 |
| 0 | 47005 | 46235 | 49505 | 50052 | 42517 | 43385 | 40341 | 40037 | 43808 | 40005 | | 41366 | 41197 |

**Table S6-5.**

| Drug  concentrations  (μg/ml) | Optical density value | | | | | | | | | | | | |
| --- | --- | --- | --- | --- | --- | --- | --- | --- | --- | --- | --- | --- | --- |
|  | MTZ | | | TDZ | | | ODZ | | | | SDZ | | |
|  | R1 | R2 | R3 | R1 | R2 | R3 | R1 | R2 | R3 | R1 | | R2 | R3 |
| 1600 | 4569 | 4633 | 4302 | 4158 | 4002 | 4246 | 4226 | 4454 | 3943 | 4061 | | 3847 | 4125 |
| 800 | 5278 | 5288 | 4806 | 4703 | 4646 | 4948 | 4860 | 4827 | 4398 | 4518 | | 4477 | 4747 |
| 400 | 5716 | 5800 | 5332 | 5278 | 4918 | 5279 | 5307 | 5426 | 4940 | 5040 | | 4858 | 5336 |
| 200 | 6162 | 6142 | 5812 | 5551 | 5676 | 5949 | 5936 | 5949 | 5523 | 5551 | | 5438 | 5915 |
| 100 | 6666 | 6613 | 6256 | 5898 | 6305 | 6454 | 6311 | 6310 | 6053 | 5654 | | 6062 | 6326 |
| 50 | 7280 | 7080 | 6927 | 5964 | 6453 | 6535 | 6730 | 6610 | 6450 | 6288 | | 6576 | 6977 |
| 25 | 6970 | 7858 | 7850 | 6244 | 6666 | 6920 | 7089 | 6828 | 6863 | 7374 | | 7539 | 7673 |
| 12.5 | 11164 | 11511 | 12767 | 6349 | 6983 | 7261 | 7303 | 6981 | 7221 | 11900 | | 12957 | 12396 |
| 6.25 | 15659 | 16654 | 17584 | 6825 | 7408 | 7221 | 8584 | 8463 | 9089 | 16166 | | 18330 | 18087 |
| 3.125 | 25154 | 24429 | 29329 | 7479 | 8230 | 7446 | 15368 | 15102 | 16470 | 24764 | | 26369 | 25628 |
| 1.563 | 35554 | 35654 | 33021 | 18505 | 18605 | 12998 | 28069 | 28119 | 25636 | 31459 | | 30128 | 31351 |
| 0.781 | 39665 | 39156 | 35598 | 41558 | 39030 | 39464 | 36443 | 40780 | 33056 | 32327 | | 29613 | 36492 |
| 0.391 | 41237 | 39280 | 38973 | 39573 | 40460 | 40499 | 38250 | 41168 | 36440 | 36695 | | 31903 | 34135 |
| 0.195 | 41722 | 42777 | 44539 | 43305 | 45579 | 39716 | 36408 | 44825 | 36172 | 42175 | | 37594 | 35089 |
| 0.098 | 45676 | 45987 | 44571 | 49115 | 45479 | 40755 | 40021 | 43459 | 36820 | 40605 | | 35700 | 34993 |
| 0.049 | 39280 | 48405 | 44972 | 50623 | 46942 | 44550 | 38749 | 40842 | 38531 | 36680 | | 42964 | 34745 |
| 0.024 | 41777 | 52098 | 46927 | 49240 | 48719 | 45313 | 40195 | 43096 | 41184 | 36666 | | 39931 | 34771 |
| 0.012 | 45108 | 48699 | 43997 | 45662 | 45705 | 43396 | 40088 | 41049 | 46131 | 38579 | | 36604 | 37185 |
| 0.006 | 47225 | 49349 | 42342 | 43760 | 43358 | 39318 | 40694 | 38953 | 44380 | 34985 | | 38256 | 34848 |
| 0 | 45624 | 50359 | 44190 | 40889 | 44450 | 44598 | 39757 | 41028 | 39556 | 34283 | | 39349 | 36265 |

**Table S6-6.**

| Drug  concentrations  (μg/ml) | Optical density value | | | | | | | | | | | | |
| --- | --- | --- | --- | --- | --- | --- | --- | --- | --- | --- | --- | --- | --- |
|  | MTZ | | | TDZ | | | ODZ | | | | SDZ | | |
|  | R1 | R2 | R3 | R1 | R2 | R3 | R1 | R2 | R3 | R1 | | R2 | R3 |
| 1600 | 4721 | 4676 | 4271 | 3892 | 3594 | 3737 | 4372 | 4357 | 3984 | 4018 | | 3804 | 4046 |
| 800 | 5693 | 5488 | 5196 | 4760 | 4676 | 4812 | 5406 | 5420 | 5201 | 5187 | | 4754 | 5343 |
| 400 | 6474 | 6432 | 6165 | 5807 | 5687 | 5821 | 6220 | 6229 | 6073 | 6063 | | 5800 | 5961 |
| 200 | 7216 | 7100 | 6807 | 6410 | 6651 | 6761 | 7051 | 7026 | 6670 | 6669 | | 6490 | 6727 |
| 100 | 7789 | 7444 | 7420 | 6983 | 7084 | 7470 | 7740 | 7680 | 7366 | 7183 | | 6160 | 7461 |
| 50 | 8224 | 7656 | 7650 | 7119 | 7485 | 7924 | 8121 | 7980 | 7728 | 7203 | | 7446 | 7661 |
| 25 | 8353 | 7972 | 7883 | 7381 | 7936 | 7951 | 8196 | 8149 | 7928 | 7437 | | 7793 | 7810 |
| 12.5 | 8295 | 7761 | 8332 | 7425 | 8175 | 8220 | 8418 | 8294 | 8293 | 7569 | | 8054 | 8012 |
| 6.25 | 8576 | 7817 | 8520 | 7260 | 8162 | 8031 | 8581 | 8452 | 8564 | 8134 | | 8621 | 8676 |
| 3.125 | 9862 | 8687 | 9622 | 7234 | 8222 | 8090 | 8378 | 8224 | 8328 | 11021 | | 9325 | 12169 |
| 1.563 | 13141 | 11750 | 11622 | 8471 | 8281 | 8629 | 10614 | 10165 | 9560 | 15004 | | 11529 | 14678 |
| 0.781 | 22078 | 19645 | 22406 | 16476 | 14858 | 11053 | 27834 | 27730 | 27802 | 31356 | | 24554 | 31046 |
| 0.391 | 30566 | 30051 | 30403 | 39247 | 38328 | 36306 | 34835 | 33734 | 33187 | 31151 | | 31090 | 33856 |
| 0.195 | 33475 | 32816 | 31329 | 36364 | 39440 | 37140 | 33910 | 29331 | 33063 | 30843 | | 31330 | 35260 |
| 0.098 | 33451 | 33289 | 29630 | 35717 | 38602 | 38547 | 34420 | 30060 | 33497 | 35441 | | 34346 | 35892 |
| 0.049 | 32393 | 29056 | 31827 | 33691 | 32463 | 36372 | 34975 | 31567 | 38031 | 30361 | | 34378 | 35200 |
| 0.024 | 31310 | 31278 | 35495 | 34019 | 37992 | 35991 | 35108 | 31936 | 34321 | 30843 | | 35029 | 34039 |
| 0.012 | 32788 | 34055 | 36544 | 35470 | 33491 | 35877 | 34675 | 32604 | 38613 | 32177 | | 33133 | 34662 |
| 0.006 | 33970 | 34676 | 33460 | 33220 | 34007 | 34648 | 34263 | 33501 | 37305 | 31367 | | 33219 | 35495 |
| 0 | 33750 | 32171 | 34471 | 30632 | 34821 | 32950 | 34446 | 30943 | 34545 | 29484 | | 32939 | 33326 |

**Table S6-7.**

| Drug  concentrations  (μg/ml) | Optical density value | | | | | | | | | | | | |
| --- | --- | --- | --- | --- | --- | --- | --- | --- | --- | --- | --- | --- | --- |
|  | MTZ | | | TDZ | | | ODZ | | | | SDZ | | |
|  | R1 | R2 | R3 | R1 | R2 | R3 | R1 | R2 | R3 | R1 | | R2 | R3 |
| 1600 | 4877 | 4802 | 4467 | 4445 | 4314 | 4552 | 4698 | 4525 | 4185 | 4142 | | 4129 | 4346 |
| 800 | 5498 | 5553 | 5027 | 4981 | 4727 | 4944 | 5063 | 4991 | 4618 | 5324 | | 4920 | 5159 |
| 400 | 8072 | 8490 | 8071 | 6002 | 5635 | 6330 | 6131 | 6231 | 5867 | 7958 | | 7814 | 7367 |
| 200 | 10730 | 10772 | 10258 | 8753 | 8590 | 8593 | 9047 | 8761 | 8614 | 10798 | | 10104 | 10720 |
| 100 | 11927 | 12051 | 11740 | 10656 | 10420 | 10542 | 11385 | 11527 | 12314 | 12478 | | 13584 | 13102 |
| 50 | 13744 | 13437 | 12695 | 11149 | 11356 | 12123 | 14449 | 13134 | 13165 | 13617 | | 14092 | 13831 |
| 25 | 15168 | 14684 | 14154 | 11845 | 12805 | 12057 | 13472 | 13928 | 15434 | 15953 | | 15251 | 14689 |
| 12.5 | 14853 | 15486 | 16001 | 12917 | 13959 | 12929 | 15906 | 16451 | 16188 | 15580 | | 15962 | 16290 |
| 6.25 | 15643 | 16924 | 18406 | 14165 | 14956 | 14621 | 15960 | 15773 | 17049 | 16173 | | 15892 | 17224 |
| 3.125 | 15022 | 15217 | 16983 | 15414 | 14367 | 14487 | 17155 | 17508 | 18010 | 15380 | | 15075 | 15132 |
| 1.563 | 21153 | 18623 | 18317 | 18571 | 18679 | 16619 | 16359 | 17375 | 18329 | 15831 | | 15616 | 15678 |
| 0.781 | 22238 | 21403 | 18568 | 21347 | 17881 | 17082 | 18208 | 19086 | 19038 | 17786 | | 16921 | 18871 |
| 0.391 | 20838 | 22261 | 23302 | 22385 | 21069 | 19170 | 16907 | 20539 | 19059 | 20655 | | 19469 | 19279 |
| 0.195 | 18786 | 21227 | 22745 | 21168 | 18833 | 18425 | 19874 | 21552 | 18375 | 20597 | | 20897 | 18964 |
| 0.098 | 21609 | 19855 | 20897 | 20357 | 20362 | 19099 | 18142 | 21540 | 19009 | 19479 | | 19500 | 17780 |
| 0.049 | 22739 | 20922 | 19249 | 21374 | 19295 | 17909 | 18457 | 20813 | 19921 | 18534 | | 19072 | 20113 |
| 0.024 | 18781 | 18907 | 18931 | 19710 | 20321 | 16221 | 17984 | 19830 | 20153 | 17294 | | 17570 | 17680 |
| 0.012 | 18387 | 19725 | 18877 | 18019 | 17576 | 15973 | 17521 | 18762 | 19536 | 17824 | | 17274 | 16688 |
| 0.006 | 20697 | 19231 | 16144 | 16407 | 16121 | 16223 | 15365 | 16154 | 19829 | 17862 | | 17169 | 18373 |
| 0 | 19805 | 19788 | 17839 | 17621 | 17265 | 17448 | 17858 | 17739 | 20087 | 17251 | | 18318 | 19482 |

**Table S6-8.**

| Drug  concentrations  (μg/ml) | Optical density value | | | | | | | | | | | | |
| --- | --- | --- | --- | --- | --- | --- | --- | --- | --- | --- | --- | --- | --- |
|  | MTZ | | | TDZ | | | ODZ | | | | SDZ | | |
|  | R1 | R2 | R3 | R1 | R2 | R3 | R1 | R2 | R3 | R1 | | R2 | R3 |
| 1600 | 4603 | 4703 | 4472 | 4352 | 4301 | 4530 | 4454 | 4517 | 4185 | 3915 | | 4181 | 4231 |
| 800 | 5187 | 4990 | 4709 | 4808 | 4847 | 4971 | 4963 | 4803 | 4684 | 4683 | | 4880 | 4980 |
| 400 | 6320 | 6554 | 6292 | 5405 | 5453 | 5495 | 5416 | 5330 | 5287 | 5950 | | 5961 | 5825 |
| 200 | 8968 | 8524 | 8872 | 6774 | 6821 | 6639 | 6469 | 6417 | 6864 | 9603 | | 9150 | 8178 |
| 100 | 9914 | 9385 | 9388 | 7855 | 8740 | 8423 | 9099 | 8796 | 8202 | 11791 | | 11940 | 10814 |
| 50 | 11462 | 11934 | 12270 | 9478 | 8914 | 9527 | 11306 | 11259 | 11509 | 13674 | | 12994 | 13093 |
| 25 | 11922 | 13021 | 13969 | 10779 | 10736 | 9383 | 12135 | 13020 | 12875 | 14214 | | 15369 | 12979 |
| 12.5 | 14701 | 12646 | 12248 | 11938 | 12264 | 11136 | 11381 | 14679 | 12448 | 20055 | | 17352 | 19846 |
| 6.25 | 16290 | 15192 | 14519 | 10209 | 12262 | 10929 | 13472 | 13513 | 16305 | 22203 | | 17580 | 16510 |
| 3.125 | 14034 | 15808 | 14172 | 14841 | 14999 | 14650 | 18759 | 16632 | 15941 | 23840 | | 20675 | 19734 |
| 1.563 | 18308 | 18881 | 16786 | 19134 | 19422 | 15961 | 22441 | 21055 | 23181 | 23765 | | 21888 | 19329 |
| 0.781 | 22109 | 20106 | 20038 | 21162 | 20923 | 21053 | 23622 | 21837 | 22506 | 24458 | | 23201 | 22697 |
| 0.391 | 22596 | 22704 | 20177 | 19535 | 19641 | 21841 | 22174 | 25238 | 20885 | 23925 | | 23477 | 22916 |
| 0.195 | 20041 | 20069 | 19787 | 22473 | 21007 | 18564 | 24362 | 25349 | 25041 | 20415 | | 20306 | 19432 |
| 0.098 | 20333 | 20381 | 22490 | 20869 | 20276 | 20541 | 24719 | 26176 | 28080 | 23358 | | 26795 | 24191 |
| 0.049 | 20675 | 20766 | 21596 | 19393 | 20755 | 17441 | 24331 | 28302 | 25347 | 22921 | | 25276 | 20054 |
| 0.024 | 20363 | 19642 | 21342 | 19774 | 21128 | 17596 | 26698 | 28163 | 29369 | 25469 | | 23767 | 20813 |
| 0.012 | 19977 | 18625 | 19714 | 19513 | 17275 | 16866 | 24487 | 23197 | 24037 | 26126 | | 24642 | 23839 |
| 0.006 | 19505 | 21946 | 18382 | 16160 | 22177 | 17194 | 22903 | 21624 | 22199 | 21020 | | 21860 | 21132 |
| 0 | 20799 | 20887 | 20399 | 20016 | 19426 | 20891 | 23908 | 21067 | 22982 | 21949 | | 22772 | 21969 |
